# *Cannabis sativa* L. alleviates loperamide-induced constipation by modulating the composition of gut microbiota in mice

**DOI:** 10.3389/fphar.2022.1033069

**Published:** 2022-12-02

**Authors:** Rong Li, Min Li, Bei Li, Wei‐Hua Chen, Zhi Liu

**Affiliations:** ^1^ Key Laboratory of Molecular Biophysics of the Ministry of Education, Department of Biotechnology, College of Life Science and Technology, Huazhong University of Science and Technology, Wuhan, China; ^2^ Key Laboratory of Molecular Biophysics of the Ministry of Education, Hubei Key Laboratory of Bioinformatics and Molecular-imaging, Center for Artificial Intelligence Biology, Department of Bioinformatics and Systems Biology, College of Life Science and Technology, Huazhong University of Science and Technology, Wuhan, China

**Keywords:** *Cannabis sativa* L., constipation, gut microbiota, *Butyricicoccus*, *Parasutterella*

## Abstract

MaZiRenWan (MZRW) is the most frequently used Traditional Chinese Medicine formula to treat chronic constipation, *Cannabis sativa* L. is regarded as a monarch drug in MZRW. However, the targets of *Cannabis sativa* L. that enhance colonic motility and improve constipation symptoms remain unknown. This study was designed to investigate the laxative effect and underlying mechanism of the water extract of *Cannabis sativa* L. (WECSL) using a loperamide-induced constipation mouse model. We found that WECSL treatment significantly improved intestinal motility and water-electrolyte metabolism, decreased inflammatory responses, prevented gut barrier damage, and relieved anxiety and depression in constipated mice. WECSL also structurally remodeled the composition of the gut microbiota and altered the abundance of bacteria related to inflammation, specifically *Butyricicoccus* and *Parasutterella*. Moreover, WECSL failed to relieve constipation symptoms following intestinal flora depletion, indicating that WECSL alleviates constipation symptoms depending on the gut microbiota. Our research provides a basis for WECSL to be further investigated in the treatment of constipation from the perspective of modern medicine.

## Introduction

Constipation, characterized by dry stools, prolonged defecation cycle, and defection difficulty, is one of the most common gastrointestinal disorders diagnosed in clinical practice, with a prevalence of 12%–19% worldwide (Bellini et al., 2021; Włodarczyk et al., 2021). Constipation decreases quality of life as patients often suffer from both physical symptoms and psychological distress (Włodarczyk et al., 2021). Treatment of constipation depends on the severity of symptoms, ranging from lifestyle modification, pharmacological management, and surgery (Bellini et al., 2021; Włodarczyk et al., 2021) Pharmacological management includes laxatives, secretagogues, serotoninergic agonists, probiotics, and prebiotics (Włodarczyk et al., 2021). Long-term use of laxatives to treat refractory constipation leads to drug overuse, which can cause dependence and adverse side effects (Tack and Muller-Lissner, 2009; Liu, 2011). Prokinetic agents, such as prucalopride, are effective in the short-term, but fail to provide long-term sustainable therapeutic efficacy (Piessevaux et al., 2015). Biofeedback therapy works effectively in limited patients, while for others the treatment is ineffective or even worsens the condition (Van Outryve and Pelckmans, 2006; Hart et al., 2011). Surgical treatment, such as colectomy, leads to serious inconveniences and reduced quality of life (Tack et al., 2011). Therefore, many patients have sought alternative medicine treatments, especially Traditional Chinese Medicine (TCM).

TCM has been used to treat various diseases for thousands of years (Zhang et al., 2019). Compared with Western medicine, TCM can treat diseases through multiple components, pathways, targets, and mechanisms. In particular, TCM has the advantages of few side effects, low recurrence rates, and significant efficacy (Wang et al., 2022). MaZiRenWan (MZRW, also known as Hemp Seed Pill) is the most frequently used TCM formula for constipation (Zhong et al., 2016). MZRW is an herbal formula that has been used to treat constipation for about 2,000 years (Huang et al., 2018). Randomized controlled clinical trials have confirmed that MZRW is a safe and effective TCM for alleviating functional constipation (FC) (Cheng et al., 2011; Zhong et al., 2019). Huang et al. performed network pharmacological analysis to predict the targets of MZRW ingredients and build a compound-target network. The representative compounds in MZRW include acetylcholine, estrogen, prostaglandin, cannabinoid, and purine, which exert the prokinetic effects of MZRW (Huang et al., 2018). *Cannabis sativa* L. (in the seed form) is regarded as a monarch drug in MZRW. *Cannabis sativa* L. contains hundreds of bioactive compounds with pharmacological activity that protect against oxidative stress and inflammation (Stasiłowicz et al., 2021; Kopustinskiene et al., 2022; Odieka et al., 2022). Lignanamides from *Cannabis sativa* L. show antioxidant and acetylcholinesterase inhibitory activities (Yan et al., 2015). However, the targets of *Cannabis sativa* L. that enhance colonic motility and improve constipation symptoms remain unknown.

The gut microbiota has been proposed to play a crucial role in TCM therapy through complicated interplay with TCM components (Xu et al., 2017). Increasing evidence indicates that gut microbiota dysbiosis could be a risk factor for chronic constipation (Ohkusa et al., 2019). Cao *et al.* found that mice administered fecal microbiota from constipated patients presented constipation symptoms (Cao et al., 2017). Many studies have indicated the promising effects of two commonly used probiotics, *Lactobacillus* and *Bifidobacteria*, in adult constipation (Ishizuka et al., 2012; Riezzo et al., 2012; Yoon et al., 2018). A recent study showed that dietary symbiotics can ameliorate constipation through modulating the gut microbiota and its metabolic function (Yang et al., 2021). Chitosan oligosaccharides were also found to attenuate constipation through structurally remodeling the gut microbial community (Zhang et al., 2021a). Therefore, we sought to investigate the laxative effect and underlying mechanism of WECSL, and whether WECSL improve constipation by modulating the gut microbiota.

## Materials and methods

### Extraction of *Cannabis sativa* L.

*Cannabis sativa* L. herb (seed) was purchased from the Tong Ren Tang herbal store. The herb was ground and crushed, and 100 g of the herb powder was first soaked in 1,000 ml water for 30 min and heated to 100°C for 20 min. The process was repeated twice, and the supernatant was filtered and collected. The water solution was concentrated in a 100 ml with a final concentration of crude herb 1 g/ml.

### Mouse model

Healthy six-week-old male C57BL/6 mice were purchased from Hubei Province Center for Disease Control and Prevention (Wuhan, China). Animals were maintained under a controlled environment with a room temperature of 23 ± 2°C under a 12/12 h light-dark cycle with access to water and a normal chow diet *ad libitum*. All animal experimental protocols were approved by the Animal Care Committee of Hubei Province and performed according to the Guide for the Care and Use of Laboratory Animals of Huazhong University of Science and Technology ([2018] IACUC Number: 2,362).

**FIGURE 1:**
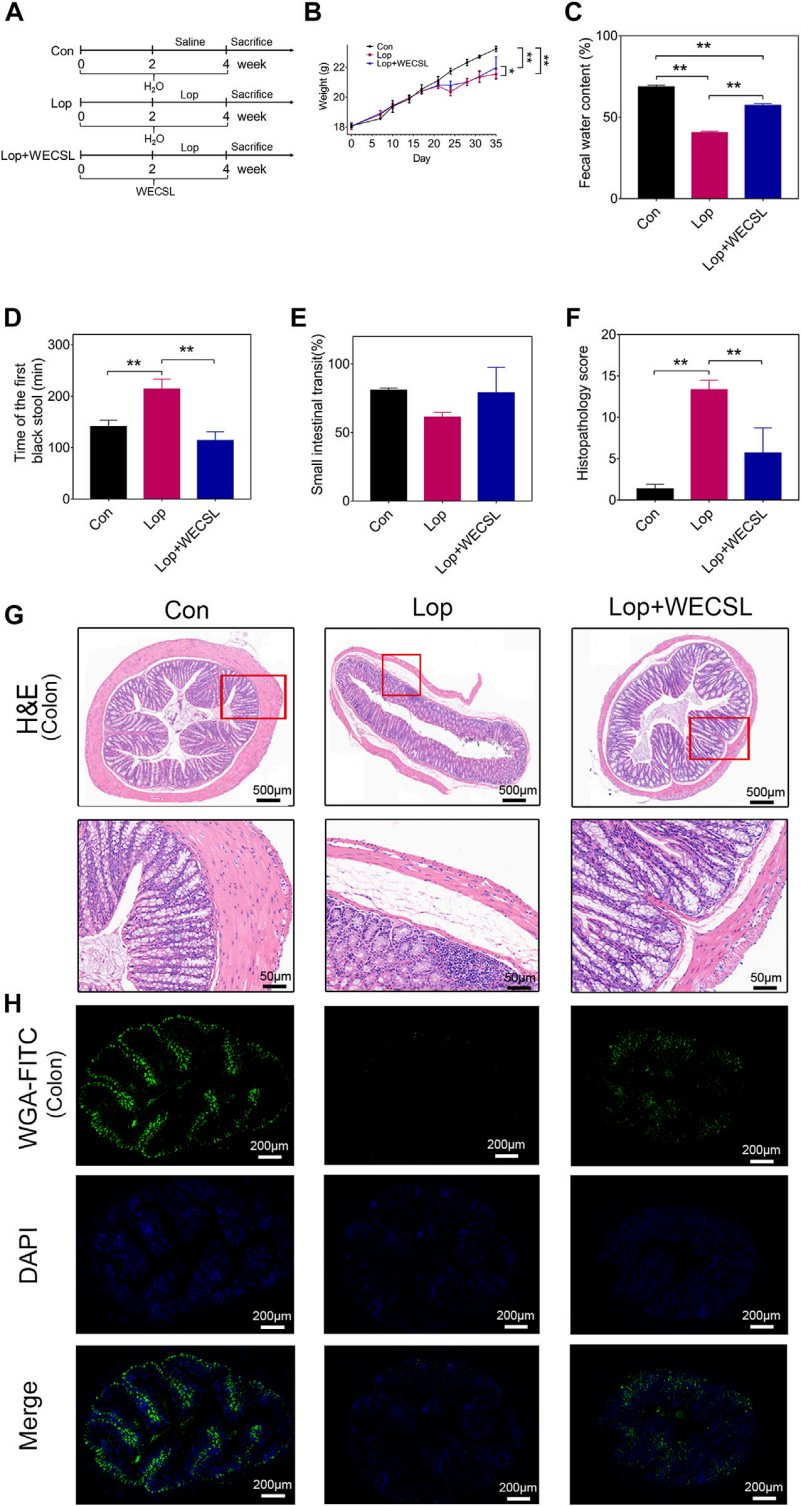
WECSL attenuated constipation symptoms in constipated mice. **(A)** Schematic diagram of the experimental interventions with WECSL in constipation mice (*n* = 8 per group). **(B)** Body weight (g). **(C)** Fecal water content. **(D)** Time of the first black stool (*n* = 5 per group). **(E)** Small intestinal transit ratio (n = 3 per group). **(F)** Histopathology score. **(G)** H&E staining of the colon. **(H)** Mucin staining with WGA-FITC in the colon. Data are presented as mean ± SD. *p < 0.05, **p < 0.01.

For functional assays, mice were acclimatized for 1 week and then randomly divided into three groups (*n* = 8 per group): blank (Con), Loperamide hydrochloride-induced constipation model (Lop), constipation mice pretreated with WECSL (Lop + WECSL) (Figure 1A). Loperamide hydrochloride (Xi’an Janssen Pharmaceutical Ltd., Xi’an, China) was solubilized in saline to a final concentration of 0.8 mg/ml. Mice were pretreated with distilled water or 100 μl WECSL (1.0 g/ml, gavage) for 2 weeks. The constipation model was developed by subcutaneously injecting mice with loperamide (10.0 mg/kg) twice a day for 2 weeks. Mice in the Con group were injected with the same volume of saline. Mice in the Lop + WECSL continued to be fed WECSL. Mice in the Con and Lop groups were given distilled water. Changes in weight, activity, and stool in all groups were recorded every 2 days during the experiment. Mouse activity was recorded as whether a mouse curled up in the corner or whether a mouse climbed to the shelf for food. The stool was measured by counting the number of feces per hour, as well as assessing fecal water content. At the experimental endpoint, serum samples, fecal samples, and colon tissues were collected. Colon tissues were flushed with PBS. For histopathological analysis, the samples were then fixed overnight in 4% paraformaldehyde (Beijing Labgic Technology Co. Ltd., Beijing, China) followed by paraffin embedding. The remaining colon tissues were snap-frozen in liquid nitrogen for subsequent experiments.

For the intestinal bacteria depletion assay, six-week-old male C57BL/6 mice were randomly divided into four groups (*n* = 8 per group): Con, Lop, Lop + antibiotic mixture treatment (Abx, 0.2 g/L ampicillin, 0.2 g/L neomycin sulfate, 0.1 g/L vancomycin and 0.2 g/L metronidazole, in drinking water), and Lop + Abx + WECSL. Mice were first pretreated with distilled water or antibiotic mixture for 2 weeks, then pretreated with distilled water or 100 μl WECSL (1.0 g/ml, gavage) for 2 weeks, followed by loperamide injection (10.0 mg/kg, twice daily) for 2 weeks to induce constipation.

### Determination of the WECSL composition by gas chromatography-mass spectrometry

One hundred microliter WECSL weighted 0.1004 g was added into a 10 ml glass tube, followed by adding 1.5 ml of 2.5% H_2_SO_4_ methanol solution (containing 0.01%BHT), 200 μl C17:0 (2 mg/ml dissolved in toluene) and 0.4 ml toluene solution. The tube was filled with nitrogen and the lid was tightened to avoid volatilization at high temperature. The tube was then placed in a water bath at 99°C for 1 h. After cooled, 1.8 ml double distilled water and 1 ml n-hexane were added and mixed. After 12 h, the supernatant was collected using a 1 ml syringe and then filtered with a 0.45 μm filter.

The fatty acid content of WECSL was assessed by Wuhan Puni Technology Co., Ltd. Using capillary chromatographic column: we observed a polydicyanopropylsiloxane polar stationary phase with a flame ionization detector. The performance was initiated at 100°C, held for 13 min; 100°C–180°C, heating rate 10°C/min, held for 6 min; 180°C–200°C, heating rate 1°C/min, held for 20 min; 200°C–230°C, heating rate 4°C/min, held for 10.5 min. The injection temperature was 270°C, and the detector temperature was 280°C. Nitrogen was used as the gas carrier at 1.0 ml/min with a split ratio of 100:1 and 1 μl of injection.

### Behavior tests

For all behavioral analyses, 2 days before the experimental endpoint, mice were transferred to the testing room to acclimatize to the ambient temperature for 1 h. The room was illuminated by adjustable lamps giving a dim light within 280 lx. All behavioral apparatus was disinfected with 70% ethanol prior to each trial as well as between trials to avoid olfactory cuing. Behavior tests, including forced swim test (FST), tail suspension test (TST), light/dark box test (LDBT), open field test (OFT), and elevated plus maze (EPM), were performed by a technician who was blind to the experimental design. Data were recorded and tracked using Smart v3.0 software (Panlab Harvard Apparatus, RWD Life Science Co., MA, United States). Details of the individual behavioral tests were conducted as follows.

FST: All mice were forced to swim for 15 min for training on the first day and swim again for 10 min on the next day. The data in the last 4 min were recorded. Locomotor activity was monitored with a video tracking system and the immobility time of each mouse was calculated. Immobility was defined as motionless between floating motions required to keep the head above water.

TST: Mice were suspended from a horizontal bar (30 cm in length) by affixing the mouse’s tail 2 cm from the tip with tape. To prevent tail climbing, we placed cylindrical plastic tubes at the base of the tail. Mice were then suspended for 6 min and video recorded. The recordings were quantified by a technician blinded to the experimental design. The total time spent in an immobile posture was measured. Immobility was defined as the absence of voluntary or escape-orientated movement.

LDBT: The apparatus consisted of a box (50 × 21 × 25 cm) that was divided into a dark and an illuminated compartment (half for each). Mice were initially placed in the dark chamber with the door open to the light. The total time spent in the light chamber and entries into the light chamber were recorded.

OFT: Mice were placed in the center of the open field container and allowed to move freely for 5 min. Activity was recorded and tracked, and the total distance traveled in the inner (23 × 23 cm central area of the OFT) and outer areas of the container were scored.

EPM: Mice were placed in the center area of the maze facing the open arms and were allowed to move freely for 6 min. Behaviors were recorded using a video tracking system. The entries into the open and closed arms and the time spent in the open arms were scored.

### Fecal water content

The collected feces from each mouse were first weighed (wet weight) and then dried at 70°C for 24 h, and the number and weight of the stool samples (dry weight) were recorded and measured. Fecal water content was calculated with the following equation: Fecal water content (%) = (wet weight - dry weight)/wet weight × 100% (Li et al., 2015).

### Intestinal transit ratio and defecation time

On day 29 after treatment, mice were fasted overnight for 12 h and then fed 300 μl of 10% activated carbon solution to observe the time of defecation of the first black stool defecation and record the intestinal transit ratio. At 30 min post oral administration, the small intestinal tissues from the pylorus to the cecum were carefully removed from three mice in each group. For each mouse, the percent of intestinal transit was calculated as the percentage of distance traveled by the charcoal meal relative to the total length of the small intestine. The following equation below was used to calculate the small intestinal transit ratio (%): Small intestinal transit ratio (%) = Transited distance by the activated carbon/Total length of the small intestine × 100%) (Li et al., 2015). The remaining five mice in each group were used to measure the time of defecation of the first black stool.

### Real-time quantitative PCR

Total RNA was extracted from 30 mg of proximal colon tissues using Trizol reagents (summer Bio, Beijing, China) according to the manufacture’s procedure. Isolated RNA was first reverse transcribed into cDNA using a first strand cDNA synthesis kit (Yeasen Bio, Shanghai, China). The cDNA was amplified with SYBR QPCR mixture (Yeasen Bio, Shanghai, China) on the BIO-RAD CFX Connect Real-time System (BioRad, CA, United States). The primers (Tsingke Biotechnology Co. Ltd., Beijing, China) used in this study are displayed in Table 1: including aquaporin 3 (*Aqp3*), AQP4, Mucin-3, epithelial sodium channel beta (*Enac-β*), *Enac-γ*, *Claudin-1*, *Occludin*, tumor necrosis factor-alpha (*Tnf-α*), interleukin-1beta (*Il-1β*), nod-like receptor pyrin domain containing 3 (*Nlrp3*), Toll-like receptor 2 (*Tlr2*), *c-kit*, stem cell factor (*Scf*), 5-hydroxytryptamine receptor 4 (*5-Ht*_*4*_ *r*), serotonin transporter (*Sert*) and *Gapdh*. PCR amplification was performed as follows: initiation at 95°C for 10 min, followed by 40 cycles of 95°C for 10 s, and 60°C for 30 s mRNA transcript levels were normalized to *Gapdh* using the 2^(−ΔΔCt)^ method.

**TABLE 1:** List of primer sequences for RT-qPCR.

Name	Forward primer (5′-3′)	Reverse primer (5′-3′)
Gapdh	AGG​TCG​GTG​TGA​ACG​GAT​TTG	TGT​AGA​CCA​TGT​AGT​TGA​GGT​CA
Aqp3	ACCCTGCCCGTGACTTTG	ACACCAGCGATGGAACCC
Aqp4	GCA​TTT​CAC​TCA​CGG​CTC​T	CTCTTGGGAACGGCACTA
Mucin-3	CGT​GGT​CAA​CTG​CGA​GAA​TGG	CGG​CTC​TAT​CTC​TAC​GCT​CTC​C
Enac-β	GGTCCTTATTGATGAGCG	TGAGAAGATGTTGGTGGC
Enac-γ	GAC​CTC​CTG​ACT​GAC​TTG​G	CCTTTCCCTTCTCGTTCT
Claudin-1	GGG​GAC​AAC​ATC​GTG​ACC​G	AGG​AGT​CGA​AGA​CTT​TGC​ACT
Occludin	TTG​AAA​GTC​CAC​CTC​CTT​ACA​GA	CCG​GAT​AAA​AAG​AGT​ACG​CTG​G
Tnf-α	GGGTGTTCATCCATTCTC	GGAAAGCCCATTTGAGT
Il-1β	GGC​TGG​ACT​GTT​TCT​AAT​GC	ATG​GTT​TCT​TGT​GAC​CCT​GA
Nlrp3	ATT​ACC​CGC​CCG​AGA​AAG​G	TCG​CAG​CAA​AGA​TCC​ACA​CAG
Tlr2	TTG​CTG​GAG​CCC​ATT​GAG​A	ATC​TTG​CGC​AGT​TTG​CAG​AA
Sert	TGG​GCG​CTC​TAC​TAC​CTC​AT	ATG​TTG​TCC​TGG​GCG​AAG​TA
C-kit	CCG​ACG​CAA​CTT​CCT​TAT​GAT	TCA​GGA​CCT​TCA​GTT​CCG​ACA
Scf	ATA​GTG​GAT​GAC​CTC​GTG​TTA	GAA​TCT​TTC​TCG​GGA​CCT​AA T
5-Ht_4_r	AGT​TCC​AAC​GAG​GGT​TTC​AGG	CAG​CAG​GTT​GCC​CAA​GAT​G

### Histopathology

The proximal colon tissues were fixed with 4% paraformaldehyde, paraffin embedded, and cut into 4 μm sections. The sections were then deparaffinized with xylene, hydrated with gradient ethanol, and rehydrated with distilled water before hematoxylin and eosin (H&E) staining (Servicebio, Wuhan, China). Histological scoring of H&E staining was done as previously reported (Schenk et al., 2007). Six parameters (mucin depletion/loss of goblet cells, crypt abscesses, epithelial erosion, hyperemia, cellular infiltration, and thickness of colonic mucosa) were scored from 0 (no alterations) to 15 (most severe). Deparaffinized and rehydrated tissue sections were first subjected to dewaxing, hydration, and antigen retrieval, then incubated with 1 μg/ml fluorescein isothiocyanate conjugated-wheat germ agglutinin (WGA-FITC; Servicebio, Wuhan, China) at 4°C overnight, followed by staining with 4′,6-diamidino-2-phenylindole (DAPI) (Invitrogen, Carlsbad, CA, United States).

### Enzyme linked immunosorbent assay (ELISA)

At the end of the experiment, serum samples from all eight mice were collected and stored at −20°C for further analysis. Alanine aminotransferase (ALT, Cat No. C010-2-1), aspartate aminotransferase (AST, Cat No. C009-2-1), motilin (MTL, Cat No. H182-1-2), and somatostatin (SS, Cat No. H092) levels were determined using ELISA kits (Jiancheng, Nanjing, China) according to the manufacturer’s instructions.

### 16S rRNA sequencing

Bacterial DNA was extracted from approximately 100 mg of stool using the QIAamp DNA Stool Mini Kit (QIAGEN, Valencia, CA). The V3-V4 variable region of 16S rRNA gene was amplified from the extracted DNA template using barcoded primers (338F 5′-ACT​CCT​ACG​GGA​GGC​AGC​AG-3′ and 806R 5′-GACTACHVGGGTWTCTAAT-3′) as follows: initiation at 95°C for 5 min, followed by 20 cycles of 95°C for 30 s, 55°C for 30 s, and 72°C for 30 s, and finally an extension at 72°C for 10 min. Sequencing was performed using an Illumina MiSeq platform (Illumina, San Diego, CA, United States) by Origingene Tech (Beijing, China) following the standard protocols.

The sequence data analyses were conducted using QIIME2 (Bolyen et al., 2019) (version 2022.2, https://qiime2.org/). Raw data were denoised to amplicon sequence variants (ASVs) by DADA2. Alpha and beta diversity analyses were done to investigate the structural variation of microbial communities across samples by R package “vegan”, and the latter was visualized using a nonmetric multidimensional scale (Ramette, 2007) (NMDS). Linear discriminant analysis (LDA) and linear discriminant analysis effect size (LEfSe) were performed to detect differentially abundant taxa across groups using the default parameters. The functional profile of a bacterial community dataset was explored using a database of phylogenetically referenced genomes (PICRUSt, Phylogenetic Investigation of Communities by Reconstruction of Unobserved States; https://www.biorxiv.org/content/10.1101/672295v2).

### Statistical analysis

**FIGURE 2:**
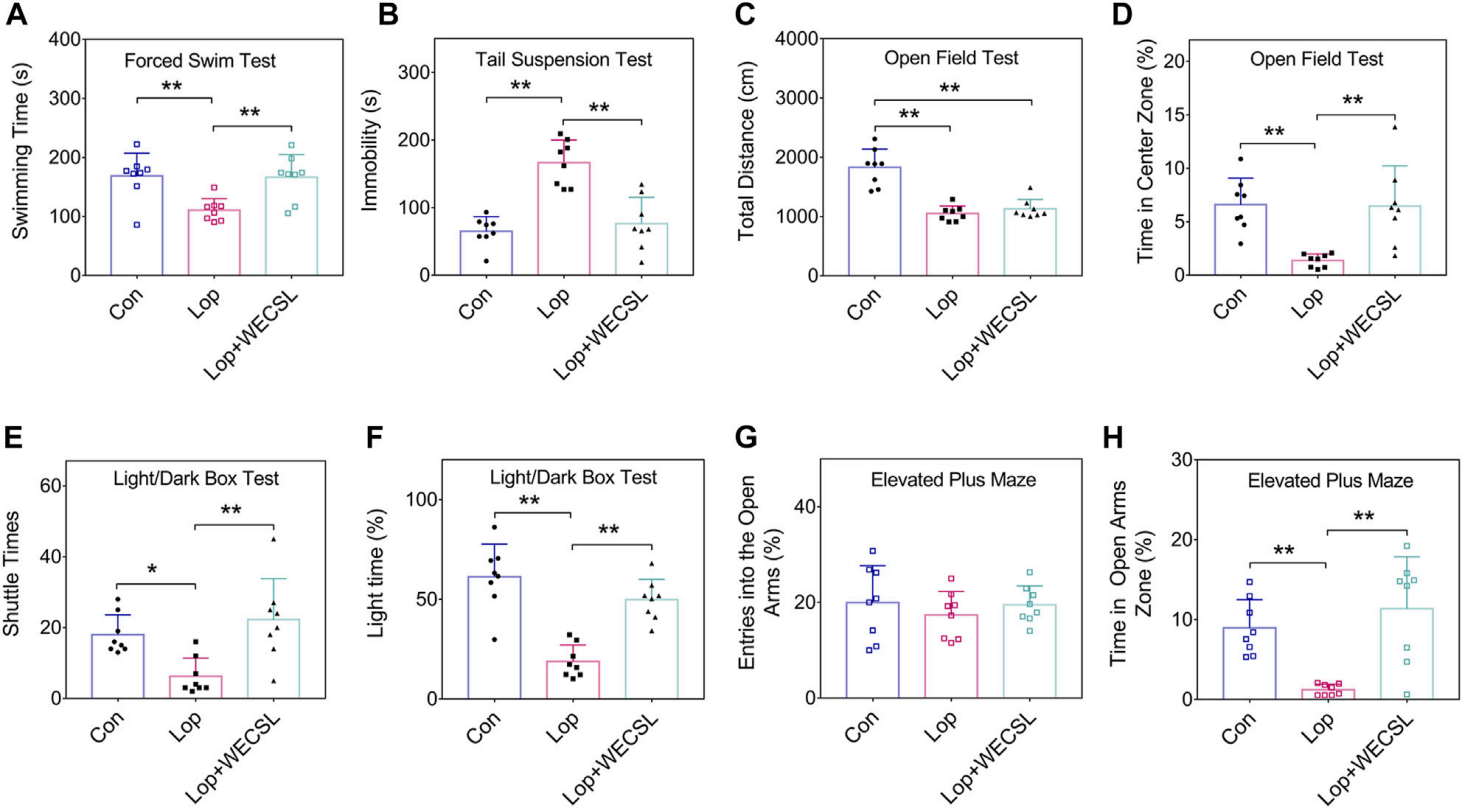
WECSL improved anxiety and depression behavioral in constipated mice. **(A)** Swimming time of mice in FST. **(B)** Immobility time of mice in TST. **(C,D)** The total distance traveled **(C)** and time stayed in the center zone **(D)** in the OFT. **(E,F)** Entries into the light chamber **(E)** and total time spent in the light chamber **(F)** in the LDBT. **(G,H)** Entries into open and closed arms **(G)** and the time spent in open arms **(H)** in the EPM. Data are presented as mean ± SD (*n* = 8 per group). *p < 0.05, **p < 0.01.

**FIGURE 3:**
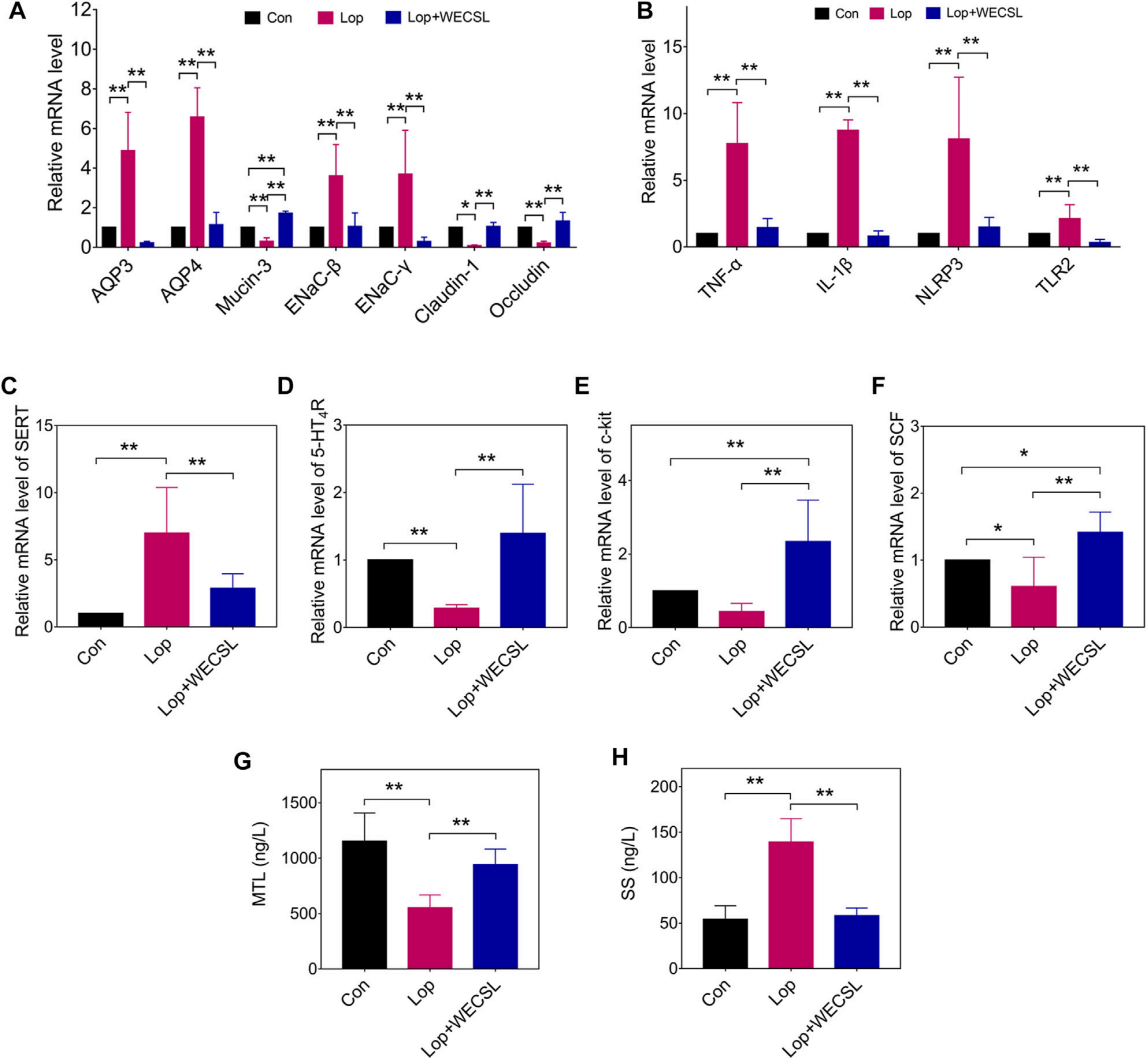
WECSL regulated disorderes of intestinal function in constipated mice. **(A)** mRNA levels of water transport-related and gut-barrier-related proteins detected using RT-qPCR. **(B)** mRNA levels of inflammatory factors, SERT **(C)**. 5-HT_4_R **(D)**, and SCF/c-kit signaling pathway proteins c-kit **(E)** and SCF **(F)** using RT-qPCR. **(G,H)** Serum MTL **(G)** and SS **(H)** levels detected using ELISA. Data are presented as mean ± SD (*n* = 8 per group). *p < 0.05, **p < 0.01.

**FIGURE 4:**
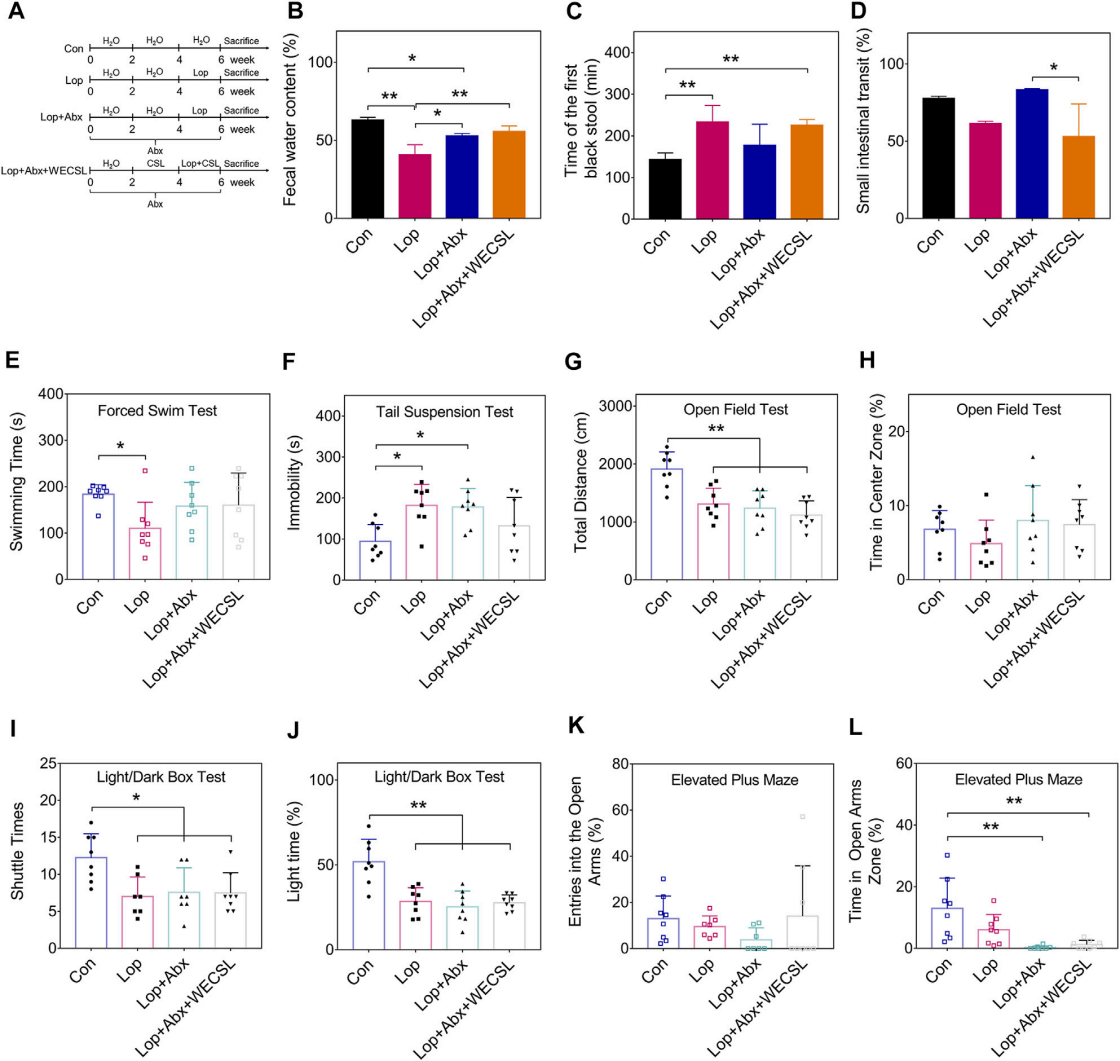
WECSL failed to improve constipation symptoms in mice following intestinal bacteria depletion. **(A)** Schematic diagram showing the animal experiments (*n* = 8 per group). **(B)** Fecal water content. **(C)** Time of the first black stool. **(D)** Small intestinal transit ratio. **(E)** Swimming time of mice in FST. **(F)** Immobility time of mice in TST. **(G,H)** The total distance traveled **(G)** and time stayed in the center zone **(H)** in the OFT. **(I,J)** Entries into the light chamber **(I)** and total time spent in the light chamber **(J)** in the LDBT. **(K,L)** Entries into open and closed arms **(K)** and the time spent in open arms **(L)** in the EPM. Data are presented as mean ± SD (*n* = 8 per group). *p < 0.05, **p < 0.01.

**FIGURE 5:**
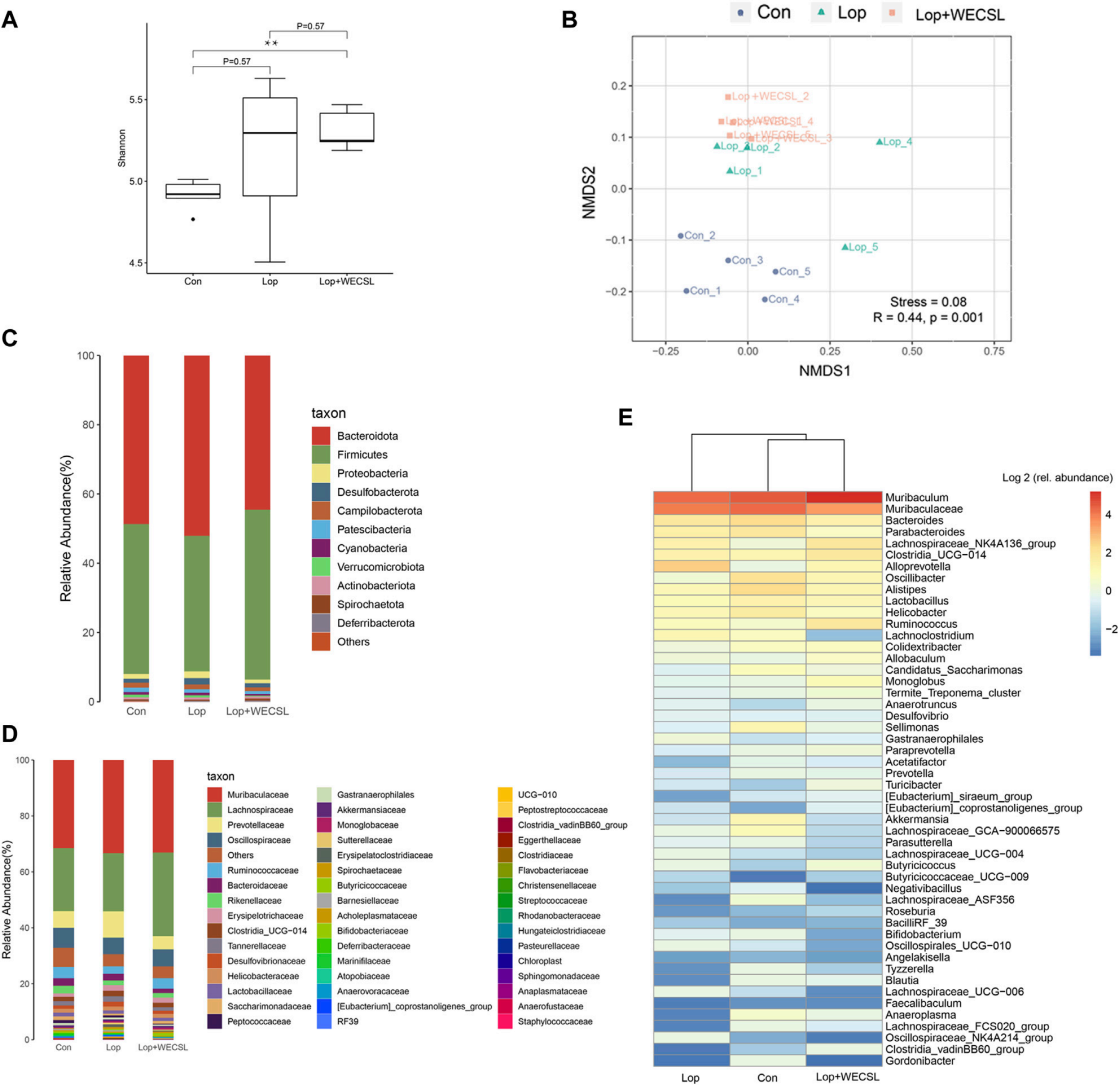
WECSL modulated gut microbiota structure in constipated mice **(A)** Alpha diversity determined using the Shannon index. **(B)** Beta diversity determined using the NMDS plot. **(C,D)** Changes in intestinal microbiota at the phylum level **(C)** and family level **(D)**. **(E)** Heap map showing the top 50 microbial community.

**FIGURE 6:**
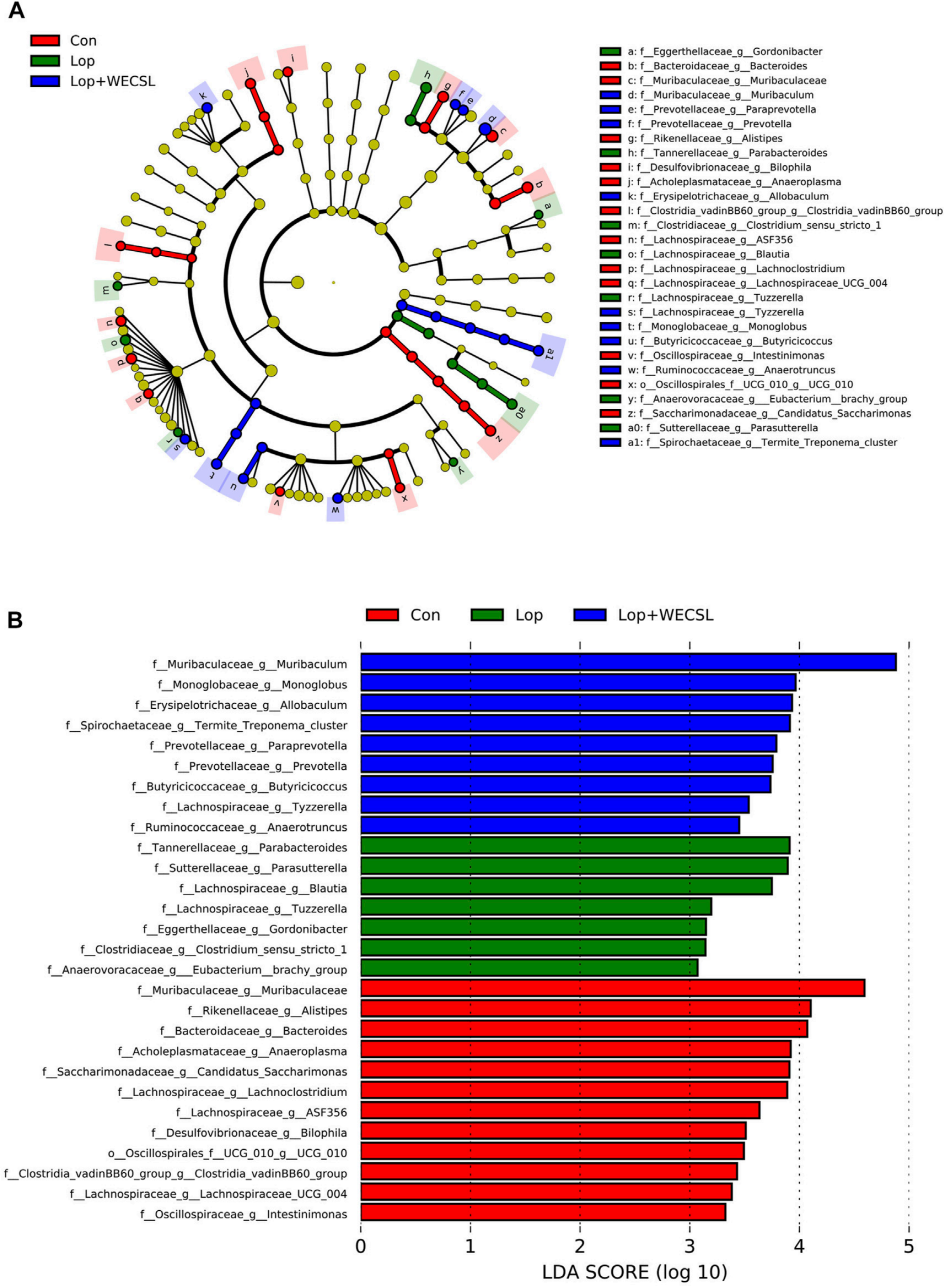
Identification of most characteristic taxa among experimental groups by linear discriminant analysis (LDA) effect size (LEfSe). **(A)** Taxonomic abundance analysis on differentially enriched taxa among three groups using LEfSe. The circle size is proportional to the relative abundance of each taxon. **(B)** Most significantly differences of intestinal bacteria taxa among three groups after LDA using a threshold score larger than 3. The bar length represents the LDA score, and color represents the enrichment direction in different groups.

**FIGURE 7:**
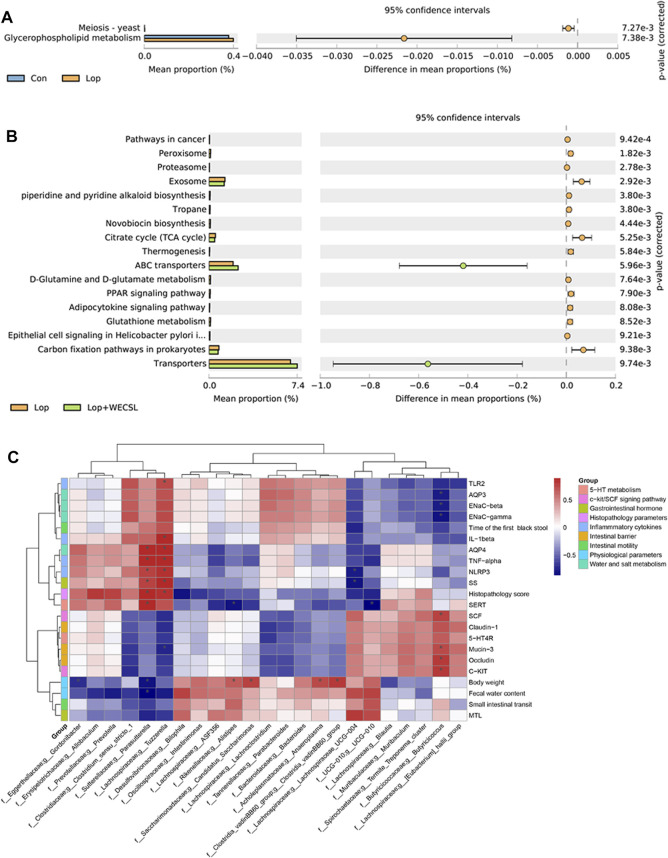
WECSL affected metabolic pathways of gut bacteria and correlation between bacterial abundance and constipation-related markers. **(A,B)** KEGG analysis of differences in the composition of metabolic pathways between Con and Lop **(A)**, Lop and Lop + WECSL **(B)**. **(C)** Spearman correlation test was used for correlation analysis, and the *p*-value was corrected using the Benjamini–Hochberg test (***FDR<0.001, **FDR<0.01, and *FDR<0.1). Red and blue colors represent significant positive correlations and negative correlations, respectively.

Data are presented as the mean ± standard deviation (SD). Statistical analysis was performed using two-way analysis of variance (ANOVA). Two-sided *t*-test was used for pairwise comparisons, and *p* values were corrected when performing multiple comparisons. The Kruskal–Wallis test was used for multiple group comparisons. Spearman correlation analysis was performed to determine the correlation between altered gut microbiota and constipation-related biological and pathological parameters and the *p*-value corrected by the Benjamini–Hochberg method (Benjamini and Hochberg, 1995) with a False Discovery Rate (FDR) threshold of 0.1 was considered statistically significant. Differences were considered significant at *p* < 0.05 or *p* < 0.01. All regular plots (Figures 1–4) were plotted using GraphPad Prism version 8.0 (La Jolla, CA, United States). All statistical analyses were performed using GraphPad Prismor and R (version 4.1.2, https://www.r-project.org/; Figures 5–7).

## Results

### Principal components of WECSL analysis by GC-MS

A total of eight constituents were chemically characterized from WECSL (Table 2) by GC-MS analysis. The total fatty acid diagrams are shown in Supplementary Material S1: Supplementary Figure S1. Qualitative analysis of WECSL showed that most of the components were linoleic acid, linolenic acid, stearic acid, palmitic acid, and oleic acid.

**TABLE 2:** Principal components of WECSL (fatty acid composition, g/100g).

Test items	Result (g)	(%)
Palmitic acid (C16:0)	0.0682	5.91
Stearic acid (C18:0)	0.039	3.38
Oleic acid (C18:1n9c)	0.174	15.08
Linoleic acid (C18:2n6c)	0.667	57.80
Arachidic acid (C20:0)	0.00702	0.61
γ- linolenic acid (C18:3n6)	0.00527	0.46
Arachidonic acid (C20:1n9)	0.00399	0.34
α-linolenic acid (C18:3n3)	0.189	16.38
Total fatty acid	1.154	

### WECSL attenuates constipation symptoms

We pretreated mice with WECSL for 2 weeks followed by loperamide to induce constipation for 2 weeks (Figure 1A). Serum AST and ALT levels showed no changes between the three groups (Supplementary Figure S2). WECSL treatment lightly increased the loperamide-induced body weight loss (Figure 1B). In the Lop + WECSL group, fecal water content was significantly higher compared to the Lop group (Figure 1C). Compared to the Lop group, the time of the first black stool was significantly shorter in the Lop + WECSL group (Figure 1D). Although WECSL treatment increased the small intestinal transit ratio compared to the Lop group, the change was not statistically different (Figure 1E).

The histopathological score analysis showed that WECSL treatment significantly decreased the pathological changes in the constipated mice (Figure 1F). The H&E staining revealed that loperamide induced fractured and uncomplete villi in the colon, and that WECSL treatment rehabilitated these effects, showing improved integrity of the colon wall and villi, increased the thickness of colon muscle layer (Figure 1G). WGA-FITC staining was used to detect colonic mucin. The results showed that colonic mucin was dramatically downregulated in the colons of the constipated mice, and WECSL treatment upregulated the expression of mucin (Figure 1H). Together, these data suggest that WECSL attenuates constipation symptoms in our mouse model.

### WECSL improves anxiety and depression behavior

Chronic constipation is often accompanied by depression and anxiety, which affect patient quality of life (Hosseinzadeh et al., 2011). We performed behavioral tests to investigate the effect of WECSL on depression and anxiety behaviors in constipated mice. As shown in Figure 2A, WECSL treatment significantly prolonged the swimming time compared with the Lop group. TST results showed that the immobility time was significantly shorter in the Lop + WECSL group compared to the Lop group (Figure 2B). WECSL treatment did not extend the total travel distance but prolonged the time in the center zone in the OFT compared to the Lop group (Figures 2C,D). LDBT results demonstrated that WECSL treatment significantly increased both the shuttle times and light time compared to the Lop group (Figures 2E,F). WECSL treatment did not increase the entries into the open arms but prolonged the time in the open arms zone in the EPM compared to the Lop group (Figures 2G,H).

### WECSL regulates disorders of intestinal function

The effects of WECSL on water-electrolyte metabolism, intestinal mucosal barrier, intestinal inflammation, and SCF/c-kit signaling pathway were assessed using RT-qPCR and ELISA. As shown in Figure 3A, overexpression of AQP3/4 and ENaC-β/γ was observed in constipated mice, all of which was decreased by WECSL treatment. WECSL statistically reversed the reduction of gut barrier-related molecules in constipated mice, including *Mucin-3*, *Claudin-1*, and *Occludin* at the mRNA level (Figure 3A). The mRNA levels of inflammatory factors (*Tnf-α, Il-1β, NLRP3*, and *Tlr2*) were also significantly increased in the constipated mice, and WECSL treatment inhibited this upregulation (Figure 3B). 5-HT, around 90% produced by enterochromaffin (EC) cells, is transported across the cell membrane by SERT. 5-HT stimulates intestinal peristalsis through promoting the release of substance P (SP) and other excitatory neurotransmitters (Mazzone et al., 2020). Tryptamine produced by bacterial decarboxylation of dietary tryptophan accelerates gastrointestinal transit by activating the epithelial G-protein-coupled receptor (GPCR) serotonin receptor-4 (5-HT_4_R) and increasing anion-dependent fluid secretion in the proximal colon (Bhattarai et al., 2018). In the constipated mice, the mRNA levels of SERT were significantly upregulated, while the mRNA levels of 5-HT_4_R were significantly downregulated. WECSL significantly downregulated SERT, but up-regulated 5-HT_4_R (Figures 3C,D). The SCF/c-kit signaling pathway has been reported to be involved in constipation (Zhang et al., 2021b). We therefore detected the mRNA levels of SCF and c-kit. The results showed that SCF/c-kit was inhibited in the constipated mice, while WECSL treatment activated the SCF/c-kit signaling pathway (Figures 3E,F). Many regulatory polypeptides, such as somatostatin (SS), motilin (MTL), gastrin (Gas), SP, endothelin (ET-1), and vasoactive intestinal peptide (VIP), also participate in the regulation of gastrointestinal transport function (McGuigan, 1978; Zhao et al., 2015). As shown in Figures 3G,H, WECSL treatment significantly increased MTL levels but significantly decreased SS levels in constipated mice.

### WECSL does not improve constipation symptoms in mice with gut microbiota depletion

WECSL contains stearic acid, palmitic acid, linoleic acid, oleic acid, linolenic acid, and other fatty acids (Table 2, Supplementary Figure S1). We explored whether WECSL also requires the participation of gut microbiota to improve constipation. Therefore, we used antibiotics to clear the gut microbiota in mice and observed whether the constipation symptoms were improved as indicated in Figure 4A. As shown in Figures 4B–D, after gut microbiota were depleted, constipation-related physiochemical parameters were slightly improved, including fecal water content, time of the first black stool, and small intestinal transit, suggesting that gut microbiota are involved in the development of constipation. However, compared to Lop + Abx mice, WECSL treatment did not increase fecal water content and shorten time of the first black stool, and small intestinal transit was even decreased. The FST, TST, OFT, and LDBT behavioral tests showed no changes between the constipated mice with intestinal bacteria depletion and the WECSL treatment group (Figures 4E–L). Together, WECSL failed to improve constipation symptoms in mice with intestinal bacteria depletion.

### WECSL modulates gut microbiota structure in constipated mice

Since the effect of WECSL on constipation was associated with intestinal bacteria, we further investigated how WECSL affects the intestinal microbiota by analyzing 16S rRNA sequencing. The alpha diversity determined by the Shannon index showed that there were significant differences between the Con group and Lop + WECSL group (Figure 5A). The beta diversity determined by NMDS showed three separated clusters, indicating different bacterial compositions among the three groups (Figure 5B). At the phylum level, mice in the Lop group had a decreased abundance of *Firmicutes* but increased amounts of *Bacteroidetes* and *Proteobacteria* compared to the Con group. WECSL treatment increased the amount of *Firmicutes* but decreased the abundance of *Proteobacteria* and *Bacteroidetes* compared to the Lop group (Figure 5C). At the family level, WECSL treatment significantly downregulated the abundance of *Prevotellaceae*, *Bacteroidaceae*, *Erysipelotrichaceae*, and *Desulfovibrionaceae*, but WECSL increased the abundance of *Lachnospiraceae*, *Ruminococcaceae*, *Lactobacillaceae*, and *Spirochaetaceae* in the constipated mice (Figure 5D). The top 50 microbial community members are shown in the heatmap in Figure 5E.

LEfSe analysis was used to compare the microbial composition and specific bacterial taxa in each experimental group. Nine dominant families in the Con group belonged to *Muribaculaceae*, *Rikenellaceae*, *Bacteroidaceae*, *Acholeplasmataceae*. *Saccharimonadaceae*, *Lachnospiraceae*, *Desulfovibrionaceae*, *Clostridia_vadinBB60_group* and *Oscillospirales*; the higher taxonomies from five key families in the Lop group were *Tannerellaceae*, *Sutterellaceae*, *Eggerthellaceae*, *Clostridiaceae* and *Anaerovoracaceae*. Additionally, *Monoglobaceae*, *Erysipelotrichaceae*, *Prevotellaceae*, *Butyricicoccaceae*, *Spirochaetaceae* and *Ruminococcaceae* were detected in the Lop + WECSL group (Figure 6A). As shown in Figure 6B, linear discriminant analysis (LDA) score demonstrated that a rich abundance of *Parabacteroides*, *Parasutterella, Blautia*, *Tuzzerella*, *Clostridium_sensu_stricto_1, Eubacterium_brachy_group* and *Gordonibacter* was detected in the Lop group, while *Muribaculaceae*, *Allobaculum*, *Alistipes*, *Bacteroides*, *Candidatus_Saccharimonas*, *Lachnoclostridium*, *Anaeroplasma*, *Lachnospiraceae__ASF356*, *Bilophila*, *Lachnospiraceae_UCG−004*, *Oscillospirales_UCG−010*, *Clostridia_vadinBB60_group* and *Intestinimonas* played major roles in the Con group; *Muribaculum*, *Termite_Treponema_cluster*, *Monoglobus*, *Prevotella*, *Paraprevotella*, *Butyricicoccus*, *Tyzzerella*, and *Anaerotruncus* were the most significant contributors in the Lop + WECSL group.

### WECSL affects metabolic pathways of gut bacteria and correlation between bacterial abundances and constipation-related markers

Next, PICRUSt analysis was carried out to estimate the impact of WECSL on metabolic pathways of intestinal bacteria in constipated mice. Based on 374 Keyoto Encyclopedia of Genes and Genomes (KEGG) pathways, 19 markedly altered pathways were screened for comparisons among experimental groups. (Figures 7A,B). Compared to the Con group, Lop significantly affected bacteria participating in meiosis-yeast and glycerophospholipid metabolism (Figure 7A). Compared to the Lop group, WECSL treatment significantly affected many pathways including peroxisome, proteasome, citrate cycle, glutathione metabolism, among others (Figure 7B). Spearman’s correlation analysis of bacterial abundances and constipation-related biomarkers was analyzed. As shown in Figure 7C, *Parasutterella*, and *Tuzzerella* were positively correlated with intestinal inflammation but negatively related to clinical presentations or mucosal barrier. In contrast, different correlations were observed between *Butyricicoccus* and physiochemical parameters (water-electrolyte metabolism and mucosal barrier) of constipated mice.

## Discussion

The pathogenesis of chronic constipation is multifactorial (Andrews and Storr, 2011). Disorders of intestinal motility and water and electrolyte transport are two main contributors to the pathogenesis (Zhao et al., 2021). These factors are jointly regulated by ion channels, AQPs, endocrine signaling, the enteric nervous system, autonomic nervous system, central nervous system, and microbiota (Zhao et al., 2021). AQPs are a family of water channel molecules that modulate water fluid homeostasis and play a pivotal role in regulating intestinal absorption, secretion, and water metabolism (Sisto et al., 2019; Zhao et al., 2021). ENaC is present on the superficial epithelial cells of the distal colon and rectum and is responsible for sodium absorption, thus maintaining ion homeostasis (Barrett, 2017). Here, we found that WECSL treatment significantly upregulated the constipation-induced decrease in mRNA levels of AQPs and protein expression of ENaC. Claudin-1, Occludin, and Mucin-3 are important intestinal tight junction proteins that maintain intestinal permeability (Moonwiriyakit et al., 2022). 5-HT is a neurotransmitter that stimulates intestinal motility and hormone secretion (Kim, 2009; Reynaud et al., 2016). Interstitial Cajal cells (ICCs) are specialized pacemaker cells that respond to the enteric motor neurotransmitters and conduct the electrical activity termed “slow wave” to coordinate the gastrointestinal motilities (Wang et al., 2018). Previous studies also found that ICCs were decreased in patients with constipation (He et al., 2000; Lyford et al., 2002). The SCF/C-kit signaling pathway is crucial in the development of ICCs. Electroacupuncture and *Cistanche deserticola* can alleviate the symptoms of constipation by targeting the SCF/C-kit signaling pathway (Zhang et al., 2021b; Kuang et al., 2022). Here, we also found that the mRNA expression of SCF/C-kit signaling proteins were dysregulated in the constipated mice, and WECSL treatment reversed these changes. These data indicate that WECSL treatment rescues intestinal motility disorders and improves water-electrolyte transportation, thus improving the symptoms of constipation and restoring abnormal behavior in constipated mice. Moreover, WECSL treatment decreased inflammation response, consistent with what has been previously reported (Dawidowicz et al., 2021; Kubiliene et al., 2021; Kopustinskiene et al., 2022; Odieka et al., 2022).

Growing evidence has indicated that constipation is strongly associated with the gut microbiota (Wang and Yao, 2021; Zhang S. et al., 2021). Many bacteria including *Bifidobacterium*, *Lactobacillus, Bacteroides, Clostridium, Ruminococcus*, and *Coprococcus* have been reported to be altered in constipation, although the findings are inconsistent (Ohkusa et al., 2019; Zhang S. et al., 2021). In the present study, we found that WECSL treatment significantly decreased the abundance of *Prevotellaceae*, *Bacteroidaceae*, *Erysipelotrichaceae*, and *Desulfovibrionaceae* but increased the abundance of *Lachnospiraceae*, *Ruminococcaceae*, *Lactobacillaceae*, and *Spirochaetaceae* in constipated mice. Besides the beneficial effect of *Lactobacillus* from *Lactobacillaceae* in patients with constipation, studies have also confirmed that intervention of *Lactobacillus* can improve intestinal mobility in pregnancy, thus improving quality of life (Liu et al., 2021; Dang et al., 2022). Both LDA and LEfSe analyses revealed that the abundance of *Ruminococcaceae* was significantly enriched after WECSL treatment. Traditionally, fatty acids are thought to have lubricant effects in treating constipation. However, there is increasing evidence to suggest that fatty acids may also have pharmacological effects in increasing bowel movement (Huang et al., 2018). For example, ricinoleic acid, a major compound in castor oil, was found to induce laxation and uterus contraction by activating EP3 receptors (Tunaru et al., 2012). These fatty acids are digested, absorbed, and transformed into active components that are useful to human health (Calder, 2015), and gut microbiota play an active role in the intermediate metabolic process (Rowland et al., 2018). WECSL is characterized by a high content of polyunsaturated fatty acids (PUFAs) and a low content of saturated fatty acids (SFAs) (Farinon et al., 2020). Linoleic acid is the most prominent PUFA in WECSL, consistent with what has been reported in the literature (Leizer et al., 2015). *Ruminococcus* and *Lactobacillus* are needed for the conversion of linoleic acid to conjugated linoleic acids (Kemp et al., 1975; Macouzet et al., 2009), which can further be metabolized into prostaglandin (Wang et al., 2006). The binding of prostaglandin to its receptor promotes gastrointestinal motility (Heeney et al., 2021). Therefore, WECSL treatment altered the composition of the gut microbiota, especially *Lactobacillaceae* and *Ruminococcaceae*, and further promoted the metabolism of WECSL, improving constipation symptoms.

Spearman’s correlation analysis revealed *Butyricicoccus* and *Parasutterella* were the two bacteria most associated with constipation. The butyrate-producing bacterium *Butyricicoccus pullicaecorum* is such a promising probiotic candidate for people suffering from inflammatory bowel disease (Geirnaert et al., 2014). Patients with inflammatory bowel disease have lower numbers of *Butyricicoccus* bacteria in their stools. Administration of *Butyricicoccus pullicaecorum* attenuates trinitrobenzenesulfonic (TNBS)-induced colitis in rats and supernatant of *Butyricicoccus pullicaecorum* cultures strengthens the epithelial barrier function by increasing the transepithelial resistance (Eeckhaut et al., 2012). Our data also showed that *Butyricicoccus* was negatively associated with the inflammatory response as well as water-electrolyte transport. These results suggest that the increase of relative abundance of *Butyricicoccus* after WECSL intervention contributes to the decrease of inflammatory response in constipated mice. Studies using a dextran sulfate sodium-induced colitis mouse model showed that *Parasutterella* was a harmful bacterium and that diet modification (Li et al., 2022) or crocetin (Dang et al., 2022) could decrease the abundance of *Parasutterella*. Maternal intake of inulin can aggravate intestinal damage and inflammation in the offspring of rats with colitis through regulating the intestinal microbiota, including increasing the abundance of *Parasutterella.* (He et al., 2022). Dietary synbiotics can ameliorate diphenoxylate-induced constipation and preserve colonic epithelial integrity through modulating gut microbiota, including reducing the relative levels of *Parasutterella* (Yang et al., 2021)*.* These studies indicate that *Parasutterella* may modulate inflammatory responses and the mucosal barrier. In the present study, *Parasutterella* was enriched in the constipation mice and WECSL treatment decreased the abundance of *Parasutterella*. Our study also showed that WECSL downregulated the inflammatory response in the constipated mice. Therefore, WECSL may decrease inflammation through reducing the abundance of *Parasutterella* while increasing the abundance of *Butyricicoccus* and then alleviating constipation.

Many methods including direct maceration, solvent extraction, soxhlex extraction, sonication, ultrasound-assisted extraction, supercritical extraction, and microwave-assisted extraction can be used to extract *Cannabis sativa* L. (Nuapia et al., 2020; Odieka et al., 2022). In this study, WECSL was used because no additional toxic substances are introduced compared to organic solvent extractions. Moreover, although serum AST and ALT levels indicated that there was no toxicity associated with WECSL treatment, it should be noted that we did not include a WECSL only treatment group in this study, and thus the data should be interpreted with caution.

TCM has been used to treat constipation for thousands of years and much progress has been achieved (Wang et al., 2022). Generally, TCM treats diseases through multiple components, targets, pathways, and mechanisms (Wang et al., 2022). The mechanisms involved in TCM treatment of constipation include warming Yang and benefiting Qi (Fuyang Tongbian Decoction, Wenyang Yiqi Prescirption and Xiaofu Tongjie Fang; all three contain *Cannabis sativa* L.), promoting blood circulation and removing blood stasis (Huayu Tongbian Decoction), nourishing Yin and moistening the intestines (Jiaweizengye Decoction, Yangyin Runchang Decoction, Buqizengye Decoction, Yangyin Runchang Prescription, Yangyin Yiqi Runchang Decoction and Tongbian Decoction; all five contain *Cannabis sativa* L.), soothing the liver and regulating Qi (Simo Decoction and Liumo Decoction), and benefiting Qi and strengthening the spleen (Zhizhu Tongbian Decoction, Jianpi Tongbian Decoction, Shutong Capsula and Yiqi Runchang Tongfu Decoction; all five contain *Cannabis sativa* L.) (Wang et al., 2022). In this study, the function of WECSL was to nourish Yin and moisten the intestines. Together, with other components, MRZW was shown to improve the condition of chronic constipation. Various compounds found in *Cannabis sativa* L. are known to provide some general health benefits that may not necessarily be disease-specific. Consumption of *Cannabis sativa* L. as a nutraceutical can therefore provide a general improvement in health.

**FIGURE 8:**
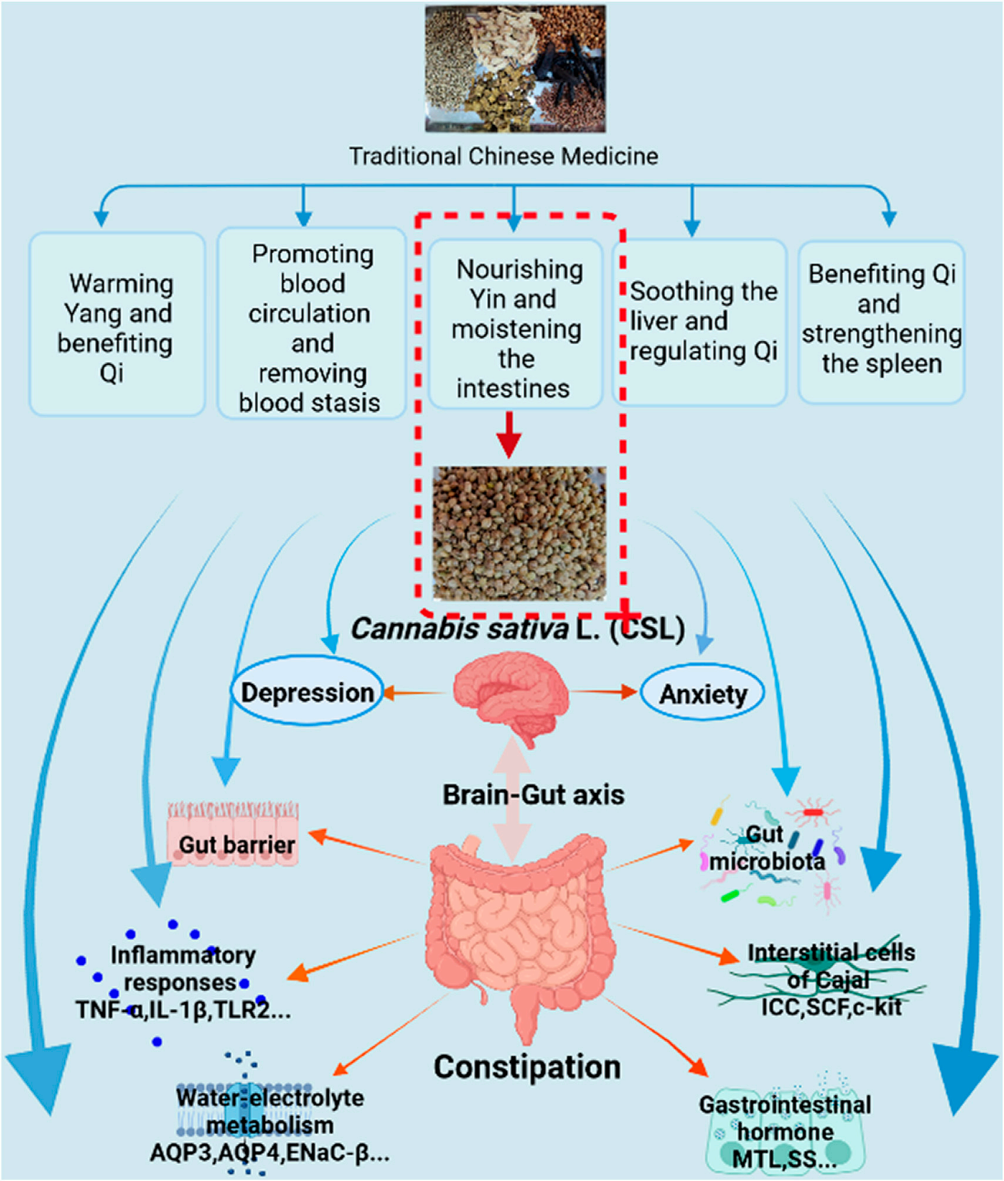
Possible underlying mechanism of *Cannabis sativa* L. for constipation treatment. The figure was made using BioRender (https://biorender.com).

In conclusion, this study demonstrated that WECSL can improve constipation symptoms, reduce anxiety and depression behaviors, and inhibit intestinal inflammation. WECSL also structurally remodeled the composition of the gut microbiota, altering the abundance of bacteria related to inflammation. Moreover, WECSL failed to relieve constipation in mice following intestinal flora depletion, indicating that WECSL alleviates loperamide-induced constipation in mice by modulating the composition of the gut microbiota, specifically *Butyricicoccus* and *Parasutterella*. The possible mechanisms of WECSL treatment on constipation are summarized in Figure 8. Our research provides a basis for WECSL to be further investigated in the treatment of constipation from the perspective of modern medicine. Constipation may be prevented and improved by targeting these possible gut bacteria. Further studies are needed to confirm the clinical therapeutic efficacy by modulating these potential gut bacteria.

## Data Availability

The datasets presented in this study can be found in online repositories. The names of the repository/repositories and accession number(s) can be found below: https://ngdc.cncb.ac.cn/search/?dbId=bioproject&q=PRJCA010873&page=1, PRJCA010873.

## Supplementary material

Supplementary Figure S1 The total fatty acid diagrams. Figure S2. Blood biochemical analysis of WECSL on mice with constipation. **(A)** Serum AST. **(B)** Serum ALT (*n*=8 per group). Data are presented as mean ± SD.

## Abbreviations

AQP3, aquaporin 3; EPM, elevated plus maze; ENaC-β, epithelial sodium channel beta; FST, forced swim test; GC-MS, Gas Chromatography-Mass Spectrometry; H&E, hematoxylin and eosin; IHC, immunohistochemistry; LDA, Linear discriminant analysis; LEfSe, linear discriminant analysis effect size; LDBT, light/dark box test; MTL, Motilin; MZRW, MaZiRenWan; NLRP3, nod-like receptor pyrin domain containing 3; OUT, operational taxonomic unit; OFT, open field test; SERT, serotonin transporter; SS, somatostatin; SCF, stem cell factor; TCM, Traditional Chinese Medicine; WECSL, water extract of *Cannabis sativa* L.; TST, tail suspension test; TNF-α, tumor necrosis factor-alpha; IL-1β, interleukin-1beta; TLR2, Toll-like receptor 2; 5-HT_4_R, 5-hydroxytryptamine receptor 4.
