# Supplementary material for: Cannabis sativa L. alleviates loperamide-induced constipation by modulating the composition of gut microbiota in mice
Source: Front Pharmacol. 2022 Dec 2;13:1033069. doi: 10.3389/fphar.2022.1033069 (PMC9755208; doi:10.3389/fphar.2022.1033069)
Supplement: Supplementary file 1 [file Table1.DOCX]

**Supplementary materials**


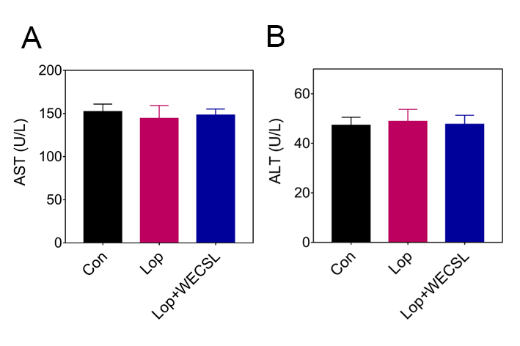


**Figure S2. Blood biochemical analysis of WECSL on constipated mice.** (A) Serum AST. (B) Serum ALT. (n=8 per group) Data are presented as mean ± SD.
