# Supplementary figures and images for: Cannabis sativa L. alleviates loperamide-induced constipation by modulating the composition of gut microbiota in mice
Source: Front Pharmacol. 2022 Dec 2;13:1033069. doi: 10.3389/fphar.2022.1033069 (PMC9755208; doi:10.3389/fphar.2022.1033069)

# Chromatogram

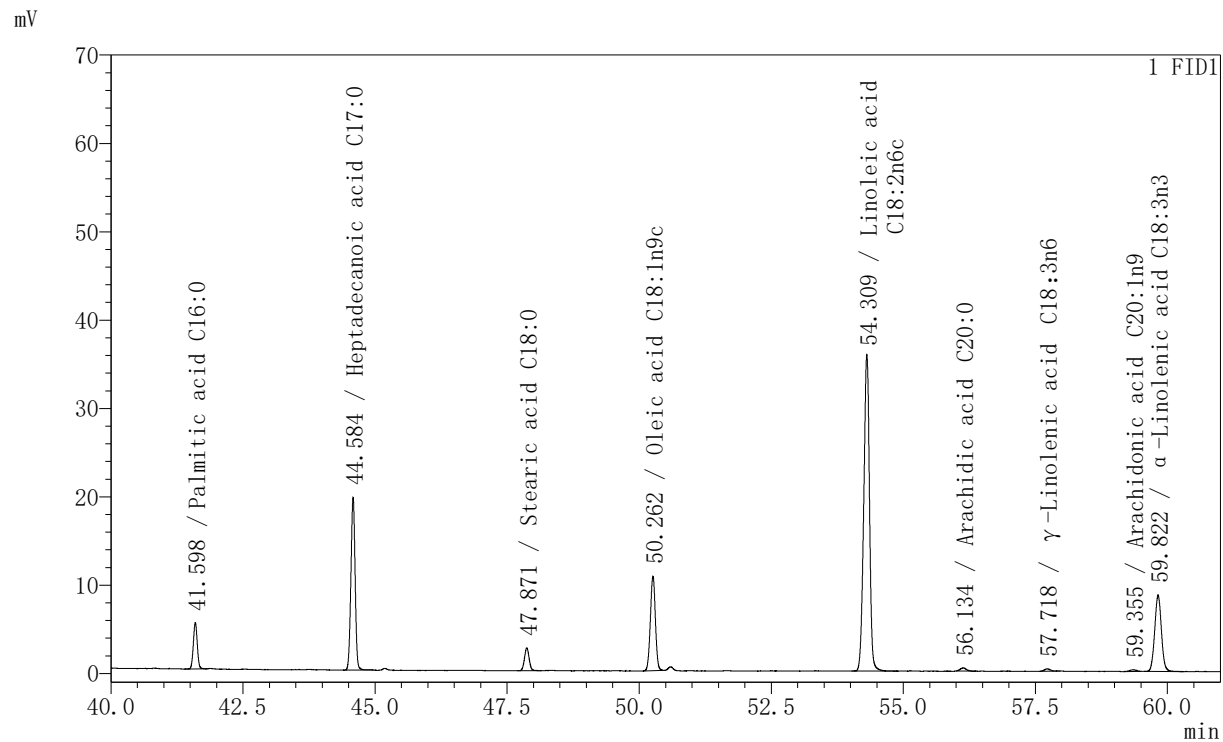

Supplement: Supplementary file 2 [file DataSheet1.PDF]
